# Intermittent preventive treatment for malaria in pregnancy in Africa: What's new, what's needed?

**DOI:** 10.1186/1475-2875-6-16

**Published:** 2007-02-16

**Authors:** Andrew Vallely, Lisa Vallely, John Changalucha, Brian Greenwood, Daniel Chandramohan

**Affiliations:** 1 National institute for Medical Research, Mwanza Centre, PO Box 1462, Mwanza, Tanzania; 2 London School of Hygiene and Tropical Medicine, Keppel Street, London, WC1E 7HT, UK

## Abstract

Falciparum malaria is an important cause of maternal, perinatal and neonatal morbidity in high transmission settings in Sub-Saharan Africa. Intermittent preventive treatment with sulphadoxine-pyrimethamine (SP-IPT) has proven efficacious in reducing the burden of pregnancy-associated malaria but increasing levels of parasite resistance mean that the benefits of national SP-IPT programmes may soon be seriously undermined in much of the region. Hence, there is an urgent need to develop alternative drug regimens for IPT in pregnancy. This paper reviews published safety and efficacy data on various antimalarials and proposes several candidate combination regimens for assessment in phase II/III clinical trials.

## Background

Falciparum malaria is an important cause of maternal anaemia, intra-uterine growth retardation, intrauterine death, stillbirth, premature delivery, low birth weight (LBW), perinatal and neonatal morbidity and mortality [1-5] and postpartum morbidity [6-8]. In sub-Saharan Africa, poor nutrition, micronutrient imbalances (particularly vitamin A, zinc, iron and folate)[1], HIV co-infection [9-12], poverty and limited access to effective primary health care and emergency obstetric services [13-15] exacerbate the impact of pregnancy-associated malaria.

In areas of high or moderate transmission, most malaria infections in pregnant women are asymptomatic and infected women do not present for treatment. In such areas, the World Health Organization recommends a combination of interventions to prevent malaria in pregnancy including insecticide-treated bednets (ITNs), intermittent preventive treatment in pregnancy (IPTp) and effective case management and treatment[16,17]. A small number of randomized controlled trials and prospective studies conducted in Kenya[18,19] and Malawi[20,21] in the 1990s demonstrated the efficacy, safety and cost-effectiveness[22] of sulphadoxine-pyrimethamine (SP) IPTp in preventing maternal anaemia and LBW. The results of these studies led to a recommendation by the World Health Organization that, in areas of medium or high malaria transmission, IPTp with SP should be given on at least two occasions following quickening[17]. Many countries in sub-Saharan Africa have subsequently introduced SP-IPTp into national malaria control programmes [23-25], but levels of coverage are still only modest in most. Although strenuous efforts are being made to increase coverage levels with SP IPTp, its effectiveness is being threatened by increasing levels of resistance to SP across Africa[23,26-32] and in SE Asia[33,34]. Thus, some authors have suggested that SP should be combined with artemisinins or with cheaper and more readily available alternatives, such as chloroquine or amodiaquine[35] to maintain the effectiveness of SP-IPTp or that other drug regimens should be used[36]. Several antimalarials, notably chloroquine, proguanil, mefloquine and proguanil-atovaquone, have been evaluated for malaria chemoprophylaxis in pregnancy[4,37-43], but few clinical trials have attempted to evaluate alternatives to SP in IPTp regimens. Three IPTp clinical trials are currently underway in Benin, Malawi and Tanzania [44-46] and will evaluate SP *vs *mefloquine[44]; SP alone *vs *SP plus artesunate[45]; and SP alone *vs *SP plus azithromycin[46] respectively. A phase III clinical trial among 900 pregnant women in Ghana concluded that amodiaquine alone or in combination with SP was effective in treating uncomplicated falciparum malaria[47], but concerns about the safety and tolerability of amodiaquine in pregnancy[48,49] and widespread resistance, particularly in East Africa[23], are likely to hinder development of amodiaquine-containing combinations for IPTp.

The future of SP for intermittent preventive treatment of malaria in pregnancy is tenuous, particularly in East and Southern Africa, and there are insufficient, reliable data on the safety and efficacy of alternative antimalarials for the prevention and treatment of malaria in pregnancy[50]. Hence, there is an urgent need to evaluate new drug combinations, particularly in areas where multi-drug resistant falciparum malaria is common[17]. This paper summarizes the current literature on this topic and suggests which antimalarial combinations should be considered for future phase II/III clinical trials among pregnant women in Africa. Key factors reviewed include evidence of safety, acceptability and favourable pharmacokinetic profile in pregnancy; the feasibility of producing fixed-dose co-formulations at costs affordable to most malaria-endemic countries; and the possibility of simple dosing regimens [51-54]. The value of combining drugs with differing molecular targets, those likely to show synergy in combination and those having comparable or disparate elimination half-lives are also discussed[55].

## Methods

### Search strategy

A two-stage, systematic search was carried out in PubMed and EMBASE using combinations of the keywords: pregnancy, malaria, antenatal, prophylaxis, intermittent, prevention, clinical trial. Alternative spellings and truncated forms were also used[56]. Articles published between 1985 and 2006 were included. Relevant references cited in review articles were also obtained. Full text articles were accessed through the WHO HINARI system designed for research institutions in developing counties[57]. Following a review of the initial search results, several antimalarial drugs were excluded from further review due to their established toxicity during pregnancy (e.g. tetracycline, doxycycline), theoretical contra-indications (e.g. primaquine, halofantrine)[54,58] or widespread resistance in the region (chloroquine, amodiaquine)[23]. The following drugs were included in a second literature search (conducted in combination with the keywords above): mefloquine; azithromycin; proguanil; chlorproguanil; dapsone; atovaquone; artesunate; artemether; dihydroartemisinin; lumefantrine; piperaquine. Trade names (e.g. Artekin^©^, Malorone^©^, Coartem^©^, Lapdap^©^, truncated forms and associated terms (e.g. macrolide, artemisinin, arte*) were used where it was felt appropriate. Manufacturers' information sheets, guidelines and reports from the World Health Organization (WHO), Medicines for Malaria Venture (MMV), Gates Malaria Partnership (GMP) and other leading organizations in the field were also scrutinized.

## Results

### Mefloquine

Mefloquine hydrochloride is a 4-quinolinemethanol derivative with a chemical structure related to that of quinine. In healthy, non-pregnant human volunteers, peak plasma concentrations of around 250 μg/L are observed 6–24 h following a single 250 mg oral administration[59]. Mefloquine has a mean elimination half-life of 2–4 weeks in healthy adults and is excreted in the bile following hepatic clearance from plasma. It can be detected in low concentrations in human breast milk following a single 250 mg dose[59]. In animal toxicity studies, mefloquine was teratogenic in mice and rats at a dose of 100 mg/kg/day and in rabbits at 80 mg/kg/day. At high doses (160 mg/kg/day), mefloquine was also embryotoxic in rabbits[59].

Mefloquine has been used alone for prophylaxis in the pre-conception period and in all trimesters of pregnancy[4,37-39,41,60] and used for the treatment of chloroquine and multidrug-resistant falciparum malaria in pregnancy, both as monotherapy[4,61-63] and in combination with other antimalarials, particularly artemisinins [64-66].

Mefloquine, at standard prophylactic doses of 250 mg/week appears safe and effective when taken before conception and during all trimesters of pregnancy[37,39,41]. In a prospective cohort of 236 pregnant travellers who took a variety of prophylactic regimens, the risk of adverse maternal and foetal outcomes among those exposed to mefloquine in the first trimester was no greater than that observed in women who took other antimalarials (chloroquine plus proguanil or sulphadoxine-pyrimethamine) or background population estimates[37]. Post-marketing surveillance by Roche Pharmaceuticals based on 1,627 reports received between 1985 and 1996 found that the risk of congenital abnormalities among women who took mefloquine for prophylaxis either before conception or at any point during pregnancy was around 4%, which was no higher than reported background population rates of around 2.5%[39]. Risk of miscarriage was unrelated to time of first exposure. Mefloquine prophylaxis 250 mg/week in the second and third trimester was also found to be safe and effective in a randomized placebo-controlled trial conducted among 339 women in Thailand[41]. Transient dizziness followed an initial 10 mg/kg loading dose, but no serious maternal adverse events, spontaneous abortions, stillbirths or neonatal deaths were reported.

However, an observational, non-comparative study among seventy-two, female US soldiers stationed in Somalia reported a risk of spontaneous abortion of 16.7% (12/72) among women who took a standard dose mefloquine prophylaxis in the first trimester[60]. One molar pregnancy was reported and 17 elective terminations carried out. No developmental abnormalities were seen among the 13 infants who were followed up for 1 year. Given the context in which the study was conducted, the small sample size and non-comparative study design, the high rate of miscarriage reported should be interpreted with caution.

There is good evidence that mefloquine is safe and effective in the treatment of falciparum malaria in pregnancy. A low dose mefloquine treatment regimen (12.5 mg/kg as single oral dose) given to 33 women in the second or third trimester in Zaria, Nigeria was well tolerated, safe and effective with no adverse maternal or foetal outcomes reported[61]. In a large prospective study in Malawi, 4,187 women who presented to an antenatal clinic with parasitaemia in the second or third trimester were randomly assigned to 1 of 4 treatment and/or prophylaxis regimens containing chloroquine or mefloquine[4]. Around 60% of women in all groups experienced minor transient symptoms such as itching or dizziness but no serious maternal adverse events were reported. The risk of spontaneous abortion was 1.2% and that of stillbirth 3.9%. No neonatal deaths or congenital abnormalities were reported and no group difference in adverse foetal outcome was observed. Mefloquine in a total treatment dose of 25 mg/kg was also safe and effective for the treatment of chloroquine-resistant falciparum malaria in 40 women enrolled in the second or third trimester in a prospective, non-comparative study conducted in Eastern Sudan[63]. Minor adverse events only were reported (e.g. 18% experienced itching and 35% nausea).

In contrast, a retrospective study in Thailand reported a borderline statistical association between mefloquine treatment during the second and third trimester of pregnancy and excess odds of stillbirth compared to women treated either with quinine or other antimalarials[62]. Among 208 women treated with 25 mg/kg mefloquine as a single treatment dose during the second and third trimester there was an excess of stillbirth (4.5%) compared to 909 women treated either with quinine (1.6%) or other antimalarials (1.4%). The stillbirth rate among women who did not take antimalarials during their pregnancy (n = 2470) was similar to that of the quinine group (1.8%). There was no group difference in the odds of spontaneous abortion or congenital abnormalities. Of the 61 stillbirths that occurred, 38 could be verified in detail including all nine cases associated with mefloquine exposure. Of these, four stillbirths resulted from intrapartum complications (prolonged labour, dystocia, eclampsia, cord prolapse), three were associated with placenta praevia and two cases were unclear ('accidental'; 'fever'). Among women exposed to all other antimalarials, 3/5 cases were of unknown cause as were 2/21 cases that occurred in women who were not exposed to antimalarials during pregnancy. After adjustment for confounders such as maternal age, gravidity, malaria attack rates and trimester of exposure, mefloquine was not significantly associated with increased odds of stillbirth if taken in the three-month prior to conception. In addition, the odds of stillbirth following mefloquine exposure in any trimester of pregnancy was not significantly different to that associated with quinine. The retrospective design, ability to trace only 72% (3,587/5,012) of women attending antenatal clinic in the observation period and biological implausibility of the associations observed suggest that these findings should be viewed within the context of other reported research, particularly clinical trials of mefloquine chemoprophylaxis, which indicate that mefloquine is safe in pregnancy.

Several studies were identified which looked at the safety and efficacy of mefloquine in combination with other antimalarials. All were conducted among women in the second or third trimester. A randomized, controlled trial with 40 subjects in Nigeria compared artemether (3.2 mg/kg intramuscularly once at entry) plus low dose mefloquine (7.5 mg/kg orally at 24, 48 h) versus artemether alone (3.2 mg/kg at entry followed by 1.6 mg/kg/day for 4 days) for the treatment of multidrug-resistant falciparum malaria[64]. No serious adverse maternal or foetal outcomes were reported. The artemether-mefloquine combination was acceptable, safe and efficacious. In another small study in Thailand, 60 women were enrolled during the second or third trimester and treated with artesunate (2 mg/kg once then 1 mg/kg twice daily for 5 days) plus mefloquine (15 mg/kg once then 10 mg/kg 6 h later) or quinine alone (10 mg/kg/day for 7 days)[67]. Fever and parasite clearance times were significantly shorter in the artesunate-mefloquine group, and fewer minor adverse events reported. No serious adverse events were observed.

A randomized, open-label clinical trial among 108 second and third trimester pregnant women in northwest Thailand compared a three-day mefloquine-artesunate regimen (MAS3; mefloquine 15 mg/kg on day 1; 10 mg/kg on day 2 plus artesunate 4 mg/kg/day on days 0, 1, 2) versus seven days treatment with quinine (Q7; 10 mg/kg three times daily for 7 days) for multidrug-resistant falciparum malaria[66]. A number of serious adverse events were reported: one maternal death, unrelated to malaria (it is not stated in which arm); two mid-trimester abortions (both in the MAS3 arm vs. none in the Q7 arm); and five neonatal deaths (three in the MAS3 arm vs. two in the Q7 arm). There were no stillbirths and no significant differences between arms in mean birthweight, proportion of LBW infants or developmental milestones at 12 months. Minor adverse events such as dizziness (87% vs. 45%; RR 1.93; 95% CI 1.14, 3.25) and tinnitus (66% vs. 17%; RR 3.93; 95% CI 1.98, 7.80) were more common in the Q7 than MAS3 group. MAS3 was more effective and better tolerated than Q7, and was associated with significantly reduced gametocyte carriage time (2.3 vs. 46.9/1000 person weeks respectively; p < 0.001). PCR adjusted recrudescence rates at 63 days were 33% in the Q7 group compared to 2% in the MAS3 group (p = 0.001).

A phase III equivalence trial comparing intermittent SP and mefloquine alone for the prevention of malaria-related morbidity in pregnancy is currently underway in Benin[44].

### Azithromycin

Azithromycin is a macrolide antibiotic derived from erythromycin which has an elimination half-life of 68 h in healthy human volunteers and is excreted largely unchanged in the bile following hepatic clearance[68]. In pregnancy, the placenta appears to act as an effective barrier to foetal exposure with limited placental transfer of macrolide antibiotics observed following maternal administration[69]. In a prospective study among 20 third trimester pregnant women, undertaken to assess maternal and transplacental pharmacokinetics of azithromycin, peak maternal serum azithromycin levels occurred within 6 h of a single 1 g oral dose and declined rapidly over the next 24 h[70]. Myometrial, adipose and placental azithromycin levels were sustained for up to 72 h post administration.

Azithromycin has been used extensively to treat sexually-transmitted and other bacterial infections during pregnancy in total treatment doses of up to 1–2 g and is considered a safe alternative for the treatment of chlamydia, mycoplasma and group B streptococci in pregnant women who are allergic to beta-lactam antibiotics [71-73].

Azithromycin is not yet licensed for use as an antimalarial agent but has shown promising activity against *P. falciparum *in vitro[74,75], in the murine malaria model[76] and in randomized controlled clinical trials [77-79]. A phase III randomized placebo-controlled clinical trial of azithromycin prophylaxis (750 mg loading dose followed by 250 mg/day for 20 weeks) versus doxycycline (100 mg/day for 20 weeks) conducted among 300 non-pregnant Indonesian adults (286/300 male) found that azithromycin was safe and well tolerated when used alone for the prevention of falciparum malaria in Northeast Papua[77]. No serious adverse events were reported. One subject developed an erythematous maculopapular rash that was probably related to azithromycin exposure and which necessitated withdrawal from the study. Prophylactic efficacy was 72% which suggests that azithromycin should be used in combination with other antimalarials rather than used alone as a first-line agent for prevention. A clinical trial among non-pregnant adults in Kenya reported similar levels of tolerability, safety and efficacy[78]. The efficacy of azithromycin in combination with other antimalarials for the treatment of uncomplicated falciparum malaria was assessed in a randomized controlled trial among 202 non-pregnant adults in Thailand[79]. A combination of artesunate 200 mg once followed by azithromycin 500 mg daily for 3 days was more effective than artesunate alone given daily for 3 days but was less effective than an artesunate plus mefloquine regimen given over 3 days. Cure rates at 28 days in the three groups were 56%, 44% and 98% respectively. No serious adverse events were reported in any of the treatment groups.

To date, no clinical trials have been conducted among pregnant women but a phase III clinical trial of intermittent SP plus azithromycin in pregnancy versus intermittent SP alone is currently underway in Malawi[46].

### Proguanil

The elimination half-life of proguanil is 12 – 21 h in healthy adult volunteers and around 40–60% of the drug is eliminated by urinary excretion. Proguanil is metabolized by a cytochrome P450 isoenzyme to an active metabolite (cycloguanil), responsible for antimalarial activity[80]. Late pregnancy and use of oral contraceptives impair formation of the active metabolite, probably due to inhibition of the metabolizing isoenzyme as a result of elevated oestrogen levels[80]. The pharmacokinetics of proguanil and cycloguanil are significantly altered in pregnant women with malaria compared to healthy volunteers and children with uncomplicated falciparum malaria[81]

Proguanil has been used extensively for malaria chemoprophylaxis in travellers, including pregnant women and children and is considered one of the safest antimalarials currently available[37,42,58,82]. A randomized controlled clinical trial in 200 antenatal clinic attenders carried out in Nigeria concluded that proguanil given in a daily dose of 100 mg daily was safe, well tolerated and effective in all trimesters of pregnancy[42]. Taken alone or in combination with chloroquine, proguanil chemoprophylaxis was similarly well tolerated and effective in a randomized prospective study among 327 antenatal clinic attenders in Muheza, Tanzania[82] and in a prospective cohort of 236 first trimester pregnant travellers[37].

### Proguanil-atovaquone

Proguanil has also been used in fixed combination with atovaquone (100 mg/250 mg respectively) in the commercially available preparation Malarone^© ^(GlaxoSmithKline). Atovaquone has an elimination half life of 2–3 days in adult volunteers and around 95% is excreted unchanged in the bile[83]. A randomized double-blind study compared mefloquine alone and proguanil-atovaquone for chemoprophylaxis in 976 non-pregnant travelers and found that both regimens were similarly effective but that the proguanil-atovaquone combination was better tolerated[84]. In an open-label randomized controlled treatment trial among 182 non-pregnant adults with multidrug-resistant falciparum malaria in Thailand, proguanil-atovaquone was more effective and better tolerated than mefloquine[85]. In a small safety and efficacy study among 26 third trimester pregnant women conducted in Thailand and Zambia, subjects presenting with acute uncomplicated falciparum malaria were given proguanil-atovaquone 100 mg/250 mg daily for 3 days[81]. Median fever clearance time was 56 h in Thailand and 52 h in Zambia; median parasite clearance times were 51 h and 24 h respectively. The 28-day cure rate was 100% at both sites. No serious maternal or foetal adverse outcomes were reported. Minor adverse events included headache, nausea and vomiting but occurred in less than 10% and were probably due to the underlying infection.

A randomized open-label clinical trial among 81 second and third trimester pregnant women in Thailand compared quinine and artesunate-atovaquone-proguanil (AAP) for the treatment of uncomplicated falciparum malaria and found that women treated with AAP had shorter fever and parasite clearance times and lower treatment failure rates (5% vs. 37%) compared to those in the quinine group[86]. The AAP combination also appeared safe and was well tolerated compared to quinine.

### Chlorproguanil-dapsone

Chlorproguanil (Lapudrine^®^) has a similar mode of action and pharmacokinetic profile to proguanil but has a slightly longer elimination half-life (around 32 h)[80]. The drug has primarily been used in fixed dose co-formulation with dapsone (diaminodiphenylsulfone), available commercially as Lapdap^© ^(GlaxoSmithKline). Despite evidence from a number of clinical trials of the safety and efficacy of this combination in the treatment of uncomplicated malaria in children[29,87,88] and pregnant women[89], a recent WHO report[80] concluded that the combination should continue to be used with caution, particularly in areas where glucose-6-phosphate dehydrogenase (G6PD) deficiency is common. Treatment trials of chlorproguanil-dapsone in pregnancy are currently underway in Tanzania[90] and in Mali[91]. A combination of chlorproguanil-dapsone plus artesunate (CDA) is also under development[92] and a multicentre phase III clinical trial comparing the efficacy and safety of CDA and artemether-lumefantrine in the treatment of uncomplicated falciparum malaria in children and adolescents is about to start in four countries in East and West Africa[93].

### Artemisinins

Artemisinin compounds (artemether, artesunate, dihydroartemisinin) are derived from sweet wormwood (*Artemisia annua*), a herb which has been used in traditional Chinese medicine for over 2000 years[55,94]. All are potent rapid-acting blood schizonticides with elimination half-lives from around 40 minutes to several hours[51].

All artemisinin analogues are associated with embryotoxicity over a narrow dose range in the rat and rabbit where embryo-lethality, late resorption and morphological abnormalities (such as abnormal development of the cardiovascular system, axial skeleton and limbs) have been reported[94,95]. There is also some evidence from animal models that artemisinins given later in pregnancy have adverse effects on foetal body weight and survival[95]. The mechanism of developmental toxicity in animals is unclear. In rats, the yolk sac is highly susceptible to artemisinin compounds[96], but recent studies in monkeys, which showed high rates of foetal loss, indicate that other mechanisms must be involved in higher mammals[97]. Published data from clinical trials in humans is very limited. Data from a total of 864 women exposed to artemisinin compounds during the second and third trimester of pregnancy have been reported to date with no evidence of drug-related adverse maternal or foetal outcome[64,66,67,86,98-100]. Published data on safety in the first trimester is limited to 50 women in The Gambia[99] and 108 in Thailand[49,100] with similar findings. None of the studies conducted to date have been specifically designed to capture excess early pregnancy loss, the key adverse pregnancy outcome that might be expected based on animal toxicity data.

Following two expert consultations in 2002, WHO recommended that artemisinins should not be used in the first trimester except in life-threatening situations where other antimalarials were not suitable, should be used with caution in the second and third trimesters and that further research be conducted to establish the pharmacokinetics of artemisinins during pregnancy, mechanism of toxicity and the relevance of animal toxicity data to humans[95]. Pharmacovigilance including post-exposure surveillance and pregnancy registers were also recommended. The pharmacokinetics of dihydroartemisinin following oral artesunate treatment was recently described in 24 second and third trimester pregnant women with uncomplicated falciparum malaria in Thailand[101].

### Artemether-lumefantrine

The only fixed-dose artemisinin combination therapy (ACT) currently available is artemether-lumefantrine (Coartem^©^/Riamet^©^), which is available at subsidized cost to malaria-endemic countries following a special pricing agreement established in 2001 between WHO and the manufacturer, Novartis[52]. Artemether-lumefantrine is now first-line treatment for uncomplicated falciparum malaria in adults and children in 19 countries in sub-Saharan Africa and 56 countries worldwide[52]. No treatment or prevention trials in pregnancy have to date been published.

### Dihydroartemisinin-piperaquine

Dihydroartemisinin is a derivative of artemisinin and the principal active metabolite of artesunate and artemether with an elimination half-life of 40–60 minutes[51]. Piperaquine is a 4-aminoquinoline structurally related to chloroquine, originally developed in China in the 1960's[102] and has an elimination half-life of 3 to 4 weeks[51]. Following the widespread introduction of piperaquine as monotherapy in China during the 1970's (including mass prophylaxis programmes in some provinces), *P. falciparum *piperaquine-resistance emerged in the mid-1990's, when efforts were then made to formulate the drug in various combinations e.g. with trimethoprim, primaquine and others[103]. Dihydroartemisinin was selected as the best partner and a fixed dose co-formulation dihyroartemisinin-piperaquine (DP; Artekin^©^/Eurartekin^©^) was developed through an international public-private partnership between Holleykin Pharmaceutical, China; Sigma-Tau Industries, Italy; the University of Oxford, UK; and the Medicines for Malaria Venture (MMV)[104]. DP has been used extensively in China, Viet Nam, Cambodia and other parts of SE Asia and shown to be safe, effective and well tolerated in the treatment of uncomplicated, multidrug-resistant falciparum malaria in children and adults[103,105-107]. Phase II/III clinical trials of DP for intermittent preventive treatment in infants (IPTi) are already underway in SE Asia and Africa[92] and the combination may soon become more widely available once international drug licensing and registration procedures have been completed. To date however, no clinical trials have been conducted among pregnant women.

### Other artemisinin combination therapies

A variety of new ACTs are currently being evaluated in phase II and III clinical trials in children and non-pregnant adults. Amodiaquine plus artesunate is being developed in fixed dose co-formulation as Coarsucam^© ^(Sanofi-Aventis). Clinical trials in Ghana[108] and Tanzania[109] have reported encouraging results with this combination in children under 5 years and a multicentre treatment trial in adults and children is about to start in Cameroon, Madagascar, Mali and Senegal.[110]. Given uncertainty about the safety of amodiaquine in pregnancy[48,49], it is unlikely that this combination will enter clinical IPTp trials in the near future. The combination may, however, be a promising candidate for evaluation in clinical trials for IPTi, particularly in West Africa where amodiaquine-SP has shown encouraging results [111-113].

The efficacy of dihydroartemisinin-mefloquine (DHA-MQ) compared to DHA alone and chloroquine alone for the treatment of uncomplicated falciparum malaria was recently described in a small study among 75 children aged 2–13 years conducted in Nigeria[114] but no published data on the use of this combination in pregnancy, evidence of clinical trials in progress or plans to develop a fixed dose formulation were identified during this review.

Pyronaridine, a Mannich-base schizontocide structurally related to the aminoacridine drug quinacrine, which exerts its antimalarial action by forming complexes with haematin[115], is being developed in fixed dose co-formulation with artesunate (PANDA)[92]. A phase II clinical trial is currently ongoing in children and adolescents in Gabon[116]. Pyronaridine has also shown promising activity in combination with dihydroartemisinin[117].

Chlorproguanil-dapsone-artesunate (CDA), artesunate-atovaquone-proguanil (AAP) and artesunate-SP are other promising ACTs under development, as described above.

## Discussion

The pharmacokinetics, dosing regimen, efficacy and safety profile of antimalarials currently used to treat and prevent falciparum malaria in adults and children are well-described. This is not the case in pregnancy where the limited data available in each of these areas makes it difficult to predict which drugs are likely to be the best candidates for treating and preventing infection. Many antimalarials are contra-indicated in pregnancy (e.g. primaquine, doxycycline, halofantrine, tafenoquine), whilst the safety of several promising candidates remains unclear[58]. In malaria-endemic countries, where antenatal HIV prevalence is high, the increased risk of serious adverse events associated with some antimalarials (e.g. severe skin reactions with SP in women with co-infection[54]) and drug interactions with anti-retrovirals used for the prevention of mother to child HIV transmission (e.g. SP interacts with nevirapine and zidovudine[118]) are also important factors to consider. Malaria in pregnancy is unique and characterized by placental sequestration of *P. falciparum*-infected erythrocytes following their adherence to placental chondroitin sulphate A (CSA)[119] and hyaluronic acid[120], which essentially allows the placenta to act as a 'privileged site' for parasite replication[36]. Pregnant women in all transmission areas have an increased susceptibility to malaria and other infections due to changes in cellular mediated immune responses [121-123], which persist into the postpartum period[6,8]. Physiological changes such as increased intravascular volume, delayed gastric emptying time, elevated oestrogen and cortisol levels and increased body fat content alter the absorption, distribution and elimination of many antimalarials during pregnancy[81]. For example the pharmacokinetics of atovaquone, proguanil, cycloguanil and dihydroartemisnin are significantly altered in pregnant women with malaria compared to healthy volunteers or adults and children with malaria[81,101]. For candidate IPTp combinations there is the added complication that there is still some debate as to how IPTp actually works: repeated short courses of antimalarials may act mainly by intermittent suppressive chemotherapy ('prophylactic effect'); by intermittent treatment of repeated infections ('treatment effect'); or by some combination of these effects[36,55]. Similar issues are also being debated about IPTi [124]. Given the early success of SP-IPTp in high transmission areas, the slow parasite clearance times and long elimination half-lives of each of the component drugs[125], it seems likely that the 'prophylactic effect' is the predominant mode of action, at least in this combination.

How then to decide which antimalarials are likely to be effective for IPT in pregnancy and which combinations should be prioritized for investigation in clinical trials (Table 1)? If the effect of IPTp is mainly prophylactic, then short-acting drugs such as quinine and the artemesinins would be expected to provide little direct benefit in asymptomatic pregnant women living in high-transmission areas since rapid parasite elimination is unnecessary. Drugs with long half-lives such as mefloquine, piperaquine or lumefantrine would, therefore, appear better choices, but monotherapy with any of these agents would be inappropriate given global patterns of drug resistance and the consensus on the need for combination therapies [51-54]. There is some evidence that combining short-acting artemisinins with longer-acting agents is likely to delay the emergence of resistance to the slowly-eliminated component[51,53,55]. Conversely, some authors advise combining drugs with similar elimination half-lives due to the risk of resistance emerging to the longer-acting drug because of it's persistence alone at sub-therapeutic levels once the rapidly eliminated drug has been excreted[54]. Combining drugs that have different molecular targets is also preferable to reduce the risk of resistance[51,54]. Acceptability, cost, possibility of a fixed-dose formulation, and a simple dosing regimen are other important considerations[53].

**Table 1:** Pharmacology of selected candidates for IPTp

**Drug**	**Mode of action**	**Elimination half life**	**Advantages/disadvantages**
**Artemether**	Inhibits falciparum sarcoplasmic-endoplasmic reticulum calcium ATPase	3 – 7 h (converted to DHA)	Appears safe in the second and third trimester Widely available in a cheap, fixed dose co-formulation with lumefantrine (Coartem^©^/Riamet^©^)
**Artemisinin**	Inhibits falciparum sarcoplasmic-endoplasmic reticulum calcium ATPase	2 – 3 h (converted to DHA)	Appears safe in the second and third trimester Fixed dose co-formulations unavailable
**Artesunate**	Inhibits falciparum sarcoplasmic-endoplasmic reticulum calcium ATPase	2 – 5 mins (converted to DHA)	Appears safe in the second and third trimester Fixed dose co-formulations unavailable
**Atovaquone**	Selective inhibitor of parasite mitochondrial metabolism	48 – 72 h	Appears safe in the third trimester Available only in fixed dose co-formulation with proguanil (Malarone^©^), which is expensive outside specific donation programmes
**Azithromycin**	Exact mode of action unknown	68 h	Safe in all trimesters where has been used extensively in STI treatment Expensive; fixed dose co-formulations unavailable
**Chlorproguanil**	Folic acid antagonist (inhibits dihydrofolate reductase)	32 h	Cheap; appears safe in the third trimester and likely to be safe earlier in pregnancy based on experience with proguanil Available only in fixed dose co-formulation with dapsone (Lapdap^©^), which WHO have recommended be used with caution in areas of high G6PD deficiency
**Dapsone**	Folic acid antagonist (inhibits dihydropteroate synthase)	31 h	Cheap; appears safe in the third trimester Available in fixed dose co-formulation with chlorproguanil (Lapdap^©^), which WHO have recommended be used with caution in areas of high G6PD deficiency
**Dihydroartemisinin (DHA)**	Inhibits falciparum sarcoplasmic-endoplasmic reticulum calcium ATPase	40 – 60 mins	Likely to be safe in the second and third trimester Available in SE Asia, China in a cheap fixed dose co-formulation with piperaquine (Artekin^©^/Eurartekin^©^)
**Lumefantrine**	Inhibits metabolism of haem within parasite acid food vacuole	4 – 6 days	Safety in children and adults established but no data available in pregnancy Widely available in a cheap [through WHO pricing agreement], fixed dose co-formulation with artemether (Coartem^©^/Riamet^©^)
**Mefloquine**	Exact mode of action unknown	2 – 4 weeks	Appears safe in the second and third trimester Expensive; fixed dose co-formulations unavailable
**Piperaquine**	Inhibits detoxification of haem	3 – 4 weeks	Safety in children and adults established but no data available in pregnancy Available in SE Asia, China in a cheap, fixed dose co-formulation with dihydroartemisinin (Artekin^©^/Eurartekin^©^)
**Proguanil**	Folic acid antagonist (inhibits dihydrofolate reductase)	12 – 21 h	Cheap; appears safe in all trimesters Available alone or in fixed dose co-formulation with atovaquone (Malarone^©^), which is expensive outside specific donation programmes
**Pyrimethamine**	Folic acid antagonist (inhibits dihydrofolate reductase)	100 h	Cheap; widely available in fixed-dose combination with sulphadoxine Increasing resistance particularly in East Africa
**Sulphadoxine**	Folic acid antagonist (inhibits dihydropteroate synthase)	200 h	Cheap; widely available in fixed-dose combination with pyrimethamine Increasing resistance particularly in East Africa

Based on these criteria, dihydroartemisinin-piperaquine appears an appropriate candidate for IPTp and should be considered for phase II/III clinical trials in second and third trimester pregnant women. The combination is relatively inexpensive (around US$ 1.00 for an adult treatment course, which is comparable to Coartem^©^)[126]; combines a very short acting artemisinin with a longer acting partner with a different mode of action; and is available in fixed-dose co-formulation. Dihydroartemisinin is the active metabolite of many artemisinin compounds, which appear to be effective, well tolerated and safe after the first trimester; the evidence for the safety of piperaquine is, however, less clear, indicating the need for preliminary phase II studies.

Artemether-lumefantrine may also be a suitable candidate: a relatively inexpensive, fixed co-formulation is available and the partner drugs have different molecular targets. The 'prophylactic effect' conferred by lumefantrine may not be sufficient however, given its relatively short half-life compared to sulphadoxine, pyrimethamine, mefloquine or piperaquine.

Mefloquine in combination with artemether or artesunate has the advantage of a similar elimination profile to dihydroartemisnin-piperaquine and the safety of such combinations in the treatment of pregnancy-associated malaria is reasonably well-established. Indeed, the extensive data available on mefloquine use in pregnancy compared to piperaquine suggest that dihydroartemisinin-mefloquine could enter phase III trials ahead of dihydroartemisinin-piperaquine. Cost and the lack of fixed dose co-formulations (although these are currently under development) are disadvantages. Combining mefloquine with the relatively long acting azithromycin is another option and likely to have additional health benefits by reducing maternal and perinatal infections, which may offset cost concerns. Sexually-transmitted infections (STIs) are common in women of childbearing age in Africa and may go undiagnosed and untreated during pregnancy[127]. Monthly azithromycin reduces STI incidence significantly in high-risk groups[128] and is safe and effective for treating gonorrhoea as well as chlamydia infection during pregnancy[71], which is associated with increased risk of stillbirth, prematurity and LBW[129]. Mefloquine-azithromycin may, therefore, represent a promising, safe and effective combination for IPTp and should be considered urgently for evaluation in phase III clinical trials.

Patterns of SP resistance vary across sub-Saharan Africa so that new artemisinin-containing combinations with SP may be appropriate in West Africa, given that these would fulfil many of the criteria above. Such combinations are, however, unlikely to succeed in East and Southern Africa, given prevailing levels of resistance.

Are different types of drug combination needed in different areas of malaria transmission? Evidence for the effectiveness of IPTp comes primarily from studies conducted in areas of intense perennial transmission (stable malaria), but there may be a case for evaluating IPTp in areas of low transmission (unstable malaria). The World Health Organization recommends that IPTp be an integral part of national malaria control programmes in high/medium perennial or seasonal transmission areas but caution that there is insufficient evidence for introducing IPTp in low transmission settings where the principal focus should be on effective case management and insecticide-treated bednets[17]. In areas of highly seasonal transmission there is some evidence to suggest that IPT given only during periods of high transmission may be effective, at least in infants[112], but no studies to date have been reported from low transmission settings such as South America or Asia, where the burden of pregnancy-associated malaria is significant, but quite different from that observed in areas of stable malaria and where the contribution of *Plasmodium vivax *infection also needs to be taken into consideration[36]. In addition, combinations found to be efficacious in sub-Saharan Africa may be of limited benefit in Asia due to their inability to prevent relapse in *P. vivax *infection[130,131].

## Authors' contributions

**AV **participated in the conception and design of the review, coordinated the literature review and drafted the manuscript.

**LV **participated in the conception and design of the review, conducted the literature search, obtained full text articles and helped draft the manuscript.

**JC **participated in the design of the review and helped draft the manuscript.

**BG **participated in the conception and design of the review, provided additional information e.g. conference proceedings and helped draft the manuscript.

**DC **conceived the study, participated in its design and coordination and helped draft the manuscript.

All authors have read and approved the final manuscript.

## Conflict of interests

The author(s) declare that they have no competing interests.
